# Letter to Editor - What makes a sustainable 24/7/365 Interventional radiology service

**DOI:** 10.1186/s42155-019-0062-4

**Published:** 2019-06-19

**Authors:** Hui Seong TEH

**Affiliations:** Ng Teng Fong General Hospital, JurongHealth Campus, National University Health System (NUHS), Jurong East Street 21, Singapore, 609606 Singapore

## Abstract

In this Letter to the Editor, the writer, shares his experience on providing a sustainable 24/7/365 interventional radiology service. Sufficient manpower resources, adequate bandwidth for quality personal and family time, consistent work processes and a cohesive team with a “whole of the patient” approach were some of the reasons emphasised.

To the Editor,

I read with interest the article “Trends and implications of 24/7 interventional radiology in a newly opened acute hospital”. Chung et al., 2018.

The authors indicate that a sufficient number of trained interventionalists with sufficient free time compensation is recommended to support a sustainable 24/7 IR clinical service in an acute hospital. I could not agree more with the authors. Burnout has high costs not only for the physician’s mental and physical health, but can impact patient health, causing adverse care events and errors as well. (Kaplan, 2018). I was the “single permanent interventional radiologist providing the dominant 24-h cover” mentioned in the article by Chung et al. Allow me to humbly share and emphasize some of the other factors that make a sustainable 24/7 IR clinical service providing satisfactory users’ experience.

We decided very early in the beginning that we need to give adequate time for our radiologists to rest and recover, to re-fresh and to re-charge and so that they are ready, well rested, re-energise and re-vitalize, for their next duty on the roster and on-call rota. Drawing brighter lines between work and time off — family, friends, outside activities, and old-fashioned daydreaming — has clear benefits for productivity, creativity, and wellness. Downtime can be essential for mental, physical, and social health. There’s an upside to downtime. (Coleman and Coleman, 2012). It reduced or eliminated a core area of stress, and that benefit results in healthier and happier employees. (Hirst, 2016). We practise dynamic and flexible roster scheduling to help us to achieve this purpose. A meaningful bandwidth for quality personal and family time for our radiologists cannot be underestimated, for a sustainable satisfactory users’ experience to the IR service.

This was a critical reason why the department was able to provide and grow the services, despite having only one single IR radiologist, during the period from before 2010 to 2014. We did increasingly more number of cases during this period of time (Table 1 and 2, Chung et al., 2018), and developed further new services, for example, IR Clinic was started.

Another important ingredient for the success of the service was the “whole of patient” approach in our practice. A successful multidisciplinary health team strives to make the most comprehensive assessment of a patient’s situation and deliver coordinated patient care to treating the whole patient (Hughes, 2018). Our team worked closely with our clinical colleagues from other departments as part of a member of the multidisciplinary team in care delivery. In areas where we did not have the resources or best expertise, we worked closely and together with our IR colleagues from other hospitals, not only those from the government public institutions but also the private services, so that we could support the needs and demands of our clinical colleagues, and our patients were not deprived of the necessary care.

Consistency in practices and processes is key to quality in care delivery, patient safety, and clinical outcome. Standardized processes or instruments help ensure consistent quality of care (Brett, 2018). Works processes, such as Standard Operating Procedures (SOP), Standard Work Instruction (SWI), and section handbook, are emphasized to ensure there is consistency in practice and processes across the entire supply chain touchpoint of IR service care continuum, for example, informed-consent process, patient preparation before a procedure, and post-procedure care.

There was also a wonderful teamwork that drove and strived the IR service. A positive workplace increases positive emotions and well-being, and achieve significantly higher levels of organizational effectiveness (Seppälä and Cameron, 2015). I thank my fellow IR nurses, and radiographers, for all the awesome supports and sacrifices during those time.

## Data Availability

Nil / Not Applicable

## Abbreviations

IR: Interventional radiology
TAT: Turnaround-time
